# Firing of Putative Dopamine Neurons in Ventral Tegmental Area Is Modulated by Probability of Success during Performance of a Stop-Change Task

**DOI:** 10.1523/ENEURO.0007-18.2018

**Published:** 2018-04-20

**Authors:** Stephen S. Tennyson, Adam T. Brockett, Nicholas W. Hricz, Daniel W. Bryden, Matthew R. Roesch

**Affiliations:** 1 Department of Psychology, University of Maryland, College Park, MD 20742; 2 Program in Neuroscience and Cognitive Science, University of Maryland, College Park, MD 20742

**Keywords:** conflict, dopamine, inhibition, neuron, rat, stop signal

## Abstract

Response inhibition, the ability to refrain from unwanted actions, is an essential component of complex behavior and is often impaired across numerous neuropsychiatric disorders such as addiction, attention-deficit hyperactivity disorder (ADHD), schizophrenia, and obsessive-compulsive disorder. Accordingly, much research has been devoted to characterizing brain regions responsible for the regulation of response inhibition. The stop-signal task, a task in which animals are required to inhibit a prepotent response in the presence of a STOP cue, is one of the most well-studied tasks of response inhibition. While pharmacological evidence suggests that dopamine (DA) contributes to the regulation of response inhibition, what is exactly encoded by DA neurons during performance of response inhibition tasks is unknown. To address this issue, we recorded from single units in the ventral tegmental area (VTA), while rats performed a stop-change task. We found that putative DA neurons fired less and higher to cues and reward on STOP trials relative to GO trials, respectively, and that firing was reduced during errors. These results suggest that DA neurons in VTA encode the uncertainty associated with the probability of obtaining reward on difficult trials instead of the saliency associated with STOP cues or the need to resolve conflict between competing responses during response inhibition.

## Significance Statement

The ability to refrain from unwanted actions, also known as response inhibition, is an essential component of complex behavior, and is impaired across numerous neuropsychiatric disorders, including addiction, attention-deficit hyperactivity disorder (ADHD), and schizophrenia. Dopamine (DA) is important for reward learning, but its role in response inhibition is less clear. For the first time, we characterized the activity of DA neurons in rats performing a response inhibition task and found that DA neurons primarily signaled information regarding the uncertainty of obtaining reward during cues and reward delivery when behavioral trials were difficult and there was a low probability of success.

## Introduction

The ability to resolve conflict between competing responses and inhibit unwanted actions, also known as cognitive control, is an essential component of complex behavior. Cognitive control is impaired in numerous neuropsychiatric disorders, including schizophrenia (Bellgrove et al., 2006; Dajani and Uddin, 2015), attention-deficit hyperactivity disorder (ADHD; Oosterlaan and Sergeant, 1998; Oosterlaan et al., 1998; Durston et al., 2009), and substance abuse disorders (Fillmore and Rush, 2002; Monterosso et al., 2005). The wide array of symptoms associated with these phenotypically distinct disorders highlights the importance of cognitive control in daily life, but also suggests that research regarding the neural mechanisms supporting conflict detection may provide useful insights into the pathologic etiology of these disorders.

Across species, and in clinical populations, a common paradigm used to test cognitive control and response inhibition is the stop-signal task (Verbruggen and Logan, 2008; Eagle and Baunez, 2010; Boecker et al., 2013). During performance of the stop-signal task, participants respond quickly to a “GO” cue (e.g., light or tone) by performing an instrumental response (e.g., button press, lever press, etc.). On GO trials (∼80% of all trials), participants develop an automatic tendency to respond quickly to the presentation of the GO cue. On “STOP” trials (∼20% of all trials), participants must inhibit this prepotent GO response when the STOP cue is presented (e.g., second light or tone). Difficulty arises from the automaticity induced by the high proportion of GO trials leading to decreased accuracy on STOP trials. In “stop-change” variants of this task, participants are not only required to inhibit their behavior on STOP trials, but also to redirect their behavior in the opposite direction (Bryden et al., 2011, 2012, 2018; Boecker et al., 2013; Bryden and Roesch, 2015). While much work has gone into the development and characterization of these tasks using pharmacological techniques, much less is known about the neural underpinnings that support this behavior.

Dopamine (DA) plays an essential role in reinforcement learning and decision-making (Roesch et al., 2007; Schultz 2013; Wood et al., 2017); however, its role in response inhibition has been incompletely studied. The use of drugs that target the DA system have yielded conflicting results ranging from improved performance on STOP trials, to altered performance on GO trials (Tannock et al., 1989; Aron et al., 2003; Bedard et al., 2003; Boonstra et al., 2005; Lijffijt et al., 2006; Eagle et al., 2008; Eagle and Baunez, 2010). Based on these results, it is difficult to parse the exact role DA plays in modulating performance on stop-signal tasks, because it remains unclear what is signaled by DA neurons during STOP tasks. It is known that separate populations of DA neurons can signal either changes in value associated with reward prediction errors or changes in the saliency of the cue, independent of its value (Matsumoto and Hikosaka 2009; Bromberg-Martin et al., 2010). That is, some DA neurons have been observed to fire more strongly for cues that predict a higher probability reward (vs cues that predict low probability reward) and also fire more strongly to delivery of unlikely reward (i.e., prediction error encoding), whereas other DA neurons increase firing to salient or alerting events independent from their value, thought to be critical for orienting and executive control (Bromberg-Martin et al., 2010).

During performance of stop-signal tasks, the activity of DA neurons may reflect reward prediction error encoding, such that DA neurons may fire less to STOP cues because they predict lower probability of reward, and fire more to successful reward delivery on STOP trials because reward delivery was less common. Alternatively, DA neurons might fire strongly to STOP cues due to their salient unexpected appearance. To test these possibilities, we recorded from putative DA neurons as rats performed our stop-change task. We found that overall DA firing was higher on GO trials during the response period, but higher on STOP trials at the time of reward. Moreover, we show that trials during which the rat was delayed in inhibiting and redirecting its behavior (i.e., response conflict), DA firing during the presentation of the STOP cue was reduced and firing during reward was more pronounced compared to trials during which response conflict was resolved more quickly. Finally, we show a correlation between activity and probability of success on difficult STOP trials such that firing was reduced after STOP cues that were preceded by multiple GO trials. Overall, these data suggest that DA firing in the ventral tegmental area (VTA) reflects the low probability of receiving a reward on STOP trials rather than a need to inhibit behavior on STOP trials or the salience associated with the low occurrence of STOP cues.

## Materials and Methods

### Animals

Four male and three female Long-Evans rats (*n* = 7; weight, 175–200 g) were obtained from Charles River Laboratories. Rats were housed on a 12/12 h light/dark schedule and all behavioral testing and recordings occurred between 9 A.M. and 2 P.M. All studies were approved by the Institutional Animal Care and Use Committee and conformed to the National Research Council Guide of the Care and Use of Laboratory Animals (2011).

### Surgical procedures and histology

Surgical procedures followed guidelines for aseptic technique. Electrodes were manufactured and implanted as in prior recording experiments (Bryden et al., 2011, 2012; Bryden and Roesch, 2015). Rats were chronically implanted with a drivable bundle of 10 25 µm in diameter FeNiCr wires (Stablohm 675, California Fine Wire) in the VTA, counterbalanced across left and right hemispheres. Four animals were implanted at 5.2 mm posterior to bregma, 0.7 mm laterally, and 7.0 mm ventral to the brain surface as in prior experiments (Roesch et al., 2007), the remaining three animals were implanted with a 5° angle pointed at the midline, with coordinates at 5.2 mm posterior to bregma, 1.4 mm laterally, and 7.5 mm ventral to the brain surface. Immediately before implantation, wires were freshly cut with surgical scissors to extend ∼1 mm beyond the cannula and electroplated with platinum (H_2_PtCl_6_; Aldrich) to an impedance of ∼300 kOhm. Cephalexin (15 mg kg^−1^, postoperative) was administered twice daily for two weeks postoperatively. After recording, rats were perfused and their brains removed and processed for histology (Roesch et al., 2006).

### Stop-change task

Recording was conducted in aluminum chambers ∼18” on each side with downward sloping walls narrowing to an area of 12” × 12” at the bottom. On one wall, a central port was located above two adjacent fluid wells. Two directional lights were located above the two fluid wells. House lights were located above the panel. Task control was implemented via computer. Port entry, licking, and well entry times were monitored by disruption of photobeams.

**Figure 1.**
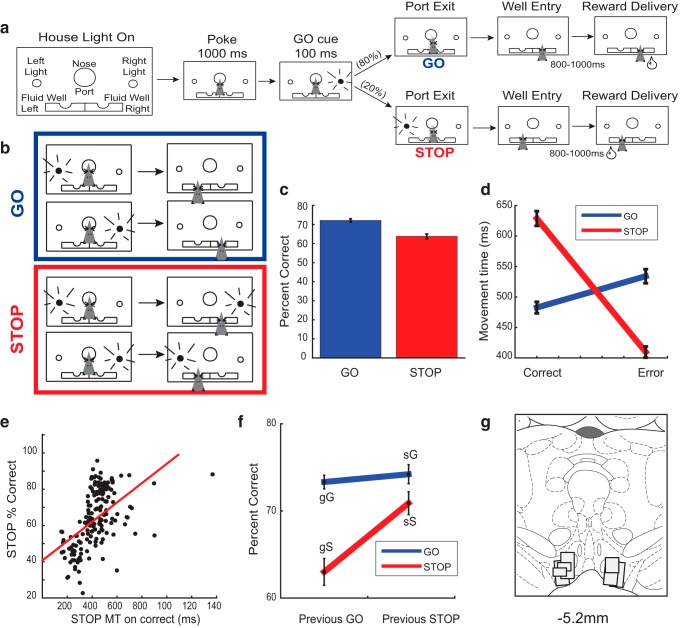
Task design and behavioral analysis. ***A***, House lights signaled the rat to nose poke and wait inside a central odor port for 1000 ms before one of two directional cue lights were illuminated for 100 ms, directing the rat to the left or right adjacent fluid well to receive fluid reward. On 20% of trials, on port exit, the opposite-sided cue light would illuminate, requiring the rat to stop the initial action and respond in the opposite direction to receive reward. After entering the correct fluid well, rats were required to hold in the well for a variable period between 800 and 1000 ms before reward delivery. ***B***, There are three trial types used in our analysis, STOP trials, GO trials, and STOP errors by two directions for each. ***C***, Bar graph shows percentage correct scores as a function of all trials. ***D***, Movement time behavior across correct and incorrect STOP (red) and GO (blue) trials. ***E***, Correlation between STOP movement time (in seconds) on correct STOP trials versus average STOP percentage correct across each session (*t* test, *p* < 0.05). ***F***, The impact of previous trial on current trial performance and conflict adaptation. Percentage correct shown across GO and STOP trials preceded by either a GO (gG, gS, left column) or STOP trial (sG, sS, right column). ***G***, Location of recording sites (Paxinos and Watson, 2007). Boxes mark the extent of recording locations based on histology.

The basic trial design is illustrated in Figure 1*A*,*B*. Each trial began by illumination of house lights that instructed the rat to nose poke into the central port. Nose poking initiated a 1000 ms pre-cue delay period. At the end of this delay, a directional light to the animal’s left or right was flashed for 100 ms. If the rat exited the port at any time before offset of the directional cue light, the trial was aborted and house lights were extinguished. On 80% of trials, presentation of the left or right light signaled the direction in which the animal could respond to obtain sucrose reward in the corresponding fluid well below. On 20% of trials, the light opposite to the location of the originally cued direction turned on either at the same time as port exit or after a stop-signal delay (0–100 ms) and remained illuminated until the behavioral response was made. These trials will be referred to as STOP trials, which were randomly interleaved with GO trials. Rats were required to stop the movement signaled by the first light and respond in the direction of the second light. On correct responding, rats were required to remain in the fluid well for a variable period between 800 and 1000 ms (pre-fluid delay) before reward delivery (10% sucrose solution). Error trials (incorrect direction) were immediately followed by the extinction of house lights and ITI onset of 4 s. Trials were presented in a pseudorandom sequence such that left and right trials were presented in equal numbers (±1 over 250 trials).

### Single-unit recordings

Procedures were the same as described previously (Bryden and Roesch, 2015). Wires were screened for activity daily; if no activity was detected, the rat was removed and the electrode assembly was advanced 40 or 80 µm. Otherwise, a session was conducted, and the electrode was advanced at the end of the session. Neural activity was recorded using four identical Plexon Multichannel Acquisition Processor Systems. Signals from electrode wires were amplified 20× by an op-amp headstage located on the electrode array. Immediately outside the training chamber, signals were passed through a differential pre-amplifier (Plexon Inc, PBX2/16sp-r-G50/16fp-G50) where single unit signals were amplified 50× and filtered at 150–9000 Hz. The single unit signals were then sent to the Multichannel Acquisition Processor box, where they were further filtered at 250–8000 Hz, digitized at 40 kHz and amplified at 1–32x. Waveforms (>2.5:1 signal-to-noise) were extracted from active channels and recorded to disk by an associated workstation with event timestamps from the behavior computer.

### DA cell identification

Neurons were screened for wide wave form and small amplitude characteristics using MATLAB as in prior experiments (Roesch et al., 2007; Takahashi et al., 2009, 2011; Takahashi and Schoenbaum, 2016; Jo et al., 2013; Sadacca et al., 2016; Park and Moghaddam, 2017; Wood et al., 2017). Wave form half duration and amplitude ratio (negative minus positive peak/sum) were calculated and clustered using k-means (Roesch et al., 2007; Takahashi et al., 2009, 2011; Takahashi and Schoenbaum, 2016; Sadacca et al., 2016). The center and variance of each cluster was computed without data from the neuron of interest, and then that neuron was assigned to a cluster if it fell within 3 SDs from the center of that cluster. If a neuron met the criteria for more than one cluster, it was not classified. This process was repeated for all neurons. Cells that increased firing to reward delivery (1 s; Wilcoxon, *p* < 0.05) and fell in the cluster with the longest half duration and smallest amplitude ratio were considered putative DA neurons (Roesch et al., 2007; Takahashi et al., 2009, 2011; Jo et al., 2013; Sadacca et al., 2016; Takahashi and Schoenbaum, 2016; Park and Moghaddam, 2017; Wood et al., 2017). We recorded from 809 VTA neurons from seven rats (1: 46 cells; 2: 141 cells; 3: 110 cells; 4: 137 cells; 5: 158 cells; 6: 120 cells; 7: 97 cells) during performance of a stop-change task (Fig. 1*A*,*B*), 85 neurons were classified as being putative DA. Of those 85 putative DA neurons, 77 were also responsive during the response epoch (i.e., nose poke exit to well entry; Wilcoxon, *p* < 0.05). A total of 475 neurons were classified as non-DA. Neurons that did not fall into one cluster or the other were excluded from analysis.

### Data analysis

Units were sorted via Offline Sorter software from Plexon Inc, using a template matching algorithm, and analyzed in Neuroexplorer and MATLAB. Activity was examined during the period between nose poke exit and well entry (response epoch), the 800 ms period following well entry (post-response epoch), and the 500 ms period following reward delivery (reward epoch). Activity in population histograms was normalized by dividing by the maximal firing rate of each neuron. Activity was averaged across direction (e.g., responding left or right) given that DA neurons are not directionally selective (Roesch et al., 2007; Wood et al., 2017). All statistical procedures were executed using raw firing rates. Unless otherwise specified, behavioral data were analyzed using a two-way ANOVA, where each datum is a session average.

## Results

### Behavior

Rats were trained to respond to left and right cue lights that directed behavior to fluid wells for reward. Our analyses examined behavior averaged over sessions rather than averaged across sessions within each rat and then across rats. Such an analysis better represents the average behavior that occurs during collection of single neuron activity that will be presented below. The behavior in this task has been replicated in several studies in rats performing the same task (Bryden et al., 2011, 2012, 2018; Bryden and Roesch, 2015). Animals exhibited significantly reduced accuracy on STOP trials compared to GO trials (*t* test: *t*_(175)_ = 6.44, *p* < 0.001; Fig. 1*C*). Rats were also significantly slower on STOP trials (Fig. 1*D*); a two-way ANOVA revealed a significant main effect of correctness (*F*_(1,643)_ = 62.07, *p* < 0.001) and a significant interaction of correctness by trial type (*F*_(1,643)_ = 161.67, *p* < 0.001). We observed no significant main effect for trial type (*F*_(1,643)_ = 1.04, *p* = 0.31). Movement times on STOP trial errors were faster than correct STOP trial types, indicating that animals failed to inhibit the initial GO response (Fig. 1*D*). Finally, rats’ performance exhibited a speed-accuracy trade-off, in that when they were slower they tended to perform better on STOP trials (Pearson’s correlation; *r* = 0.52; *p* < 0.001; Fig. 1*E*).

Performance on the current trial depended on the difficulty of the previous trial type. To determine the effects of previous trial type on the current trial’s performance, percentage correct was analyzed across all possible combinations of current and previous trials [i.e., when STOP preceded STOP (sS); GO preceded STOP (gS); GO preceded GO (gG); STOP preceded GO (sG)]. STOP trials after GO trials were more difficult as evidenced by worse performance (Fig. 1*F*). A two-way ANOVA revealed a significant main effect of the previous trial type (*F*_(1,651)_ = 13.01, *p* < 0.001), a significant main effect of the current trial type (*F*_(1,651)_ = 31.40, *p* < 0.001), and a significant interaction between the previous and current trial type (*F*_(1,651)_ = 8.26, *p* = 0.004), demonstrating that percentage correct was significantly lower on STOP trials preceded by GO trials and that rats demonstrated conflict adaptation such that they were more accurate on STOP trials immediately following STOP trials.

### Putative DA neurons show lower firing to STOP cues during the response period but higher for STOP trials at the time of reward

**Figure 2.**
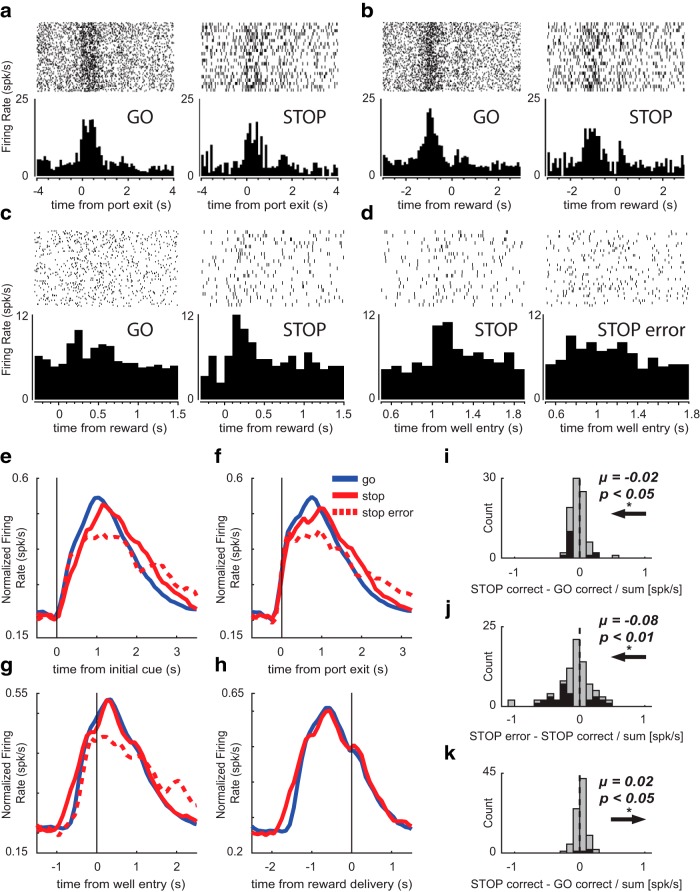
Putative DA firing was higher for GO trials during the response period but higher for STOPs at the time of reward. ***A***, Single-cell example of a putative DA neuron aligned to port exit that fired more for GO trials (left) than STOP trials (right). ***B***, The same single-cell example from ***A*** aligned to reward delivery, showing higher firing to reward on STOP trials (right) compared to GO trials (left). ***C***, The same single-cell example from ***A***, ***B*** aligned to reward delivery and zoomed in on the reward response, showing higher firing on STOP trials (right) compared to GO trials (left). ***D***, The same single-cell example from ***A***, ***B*** zoomed in on the response following well entry, showing higher firing on correct STOP trials (left) compared to STOP errors (right). ***E–H***, Population average histograms of putative DA neurons plotted over trial time for GO (blue), STOP (red), and STOP error (dashed) trials while aligning to multiple events (*n* = 85). Activity is aligned to initial GO cue (***E***), port exit (***F***), well entry (***G***), and reward delivery (***H***). ***E***, At the time of the initial cue, firing rate increases rapidly for all trial types. ***F***, During the response epoch (the period from port exit to well entry, when the STOP cue was illuminated on STOP trials), firing was significantly higher on GO trials compared to STOP trials. ***G***, During the post-response epoch (the 800 ms period following well entry), firing was significantly lower on STOP errors compared to STOP corrects. ***H***, During the reward epoch (500 ms period after reward delivery), firing was significantly higher on STOP trials than GO trials. Error trials are excluded since reward is not delivered on errors. ***I***–***K***, Population average distributions for significant effects described above. Arrows depict direction of distribution shift for significant effects. Black bars represent the number of neurons that showed a significant difference between GO and STOP corrects (***I***, ***K***) and correct and incorrect STOP trials (***J***; Wilcoxon, *p* < 0.05). Distributions are determined to be significantly different from zero via Wilcoxon. ***I***, ***K***, Firing rate during the response and reward epoch were compared during correct STOPs and GOs by computing a trial type index (STOP correct – GO correct/STOP correct + GO correct). ***J***, Firing rate during the post-response epoch was compared in correct and incorrect STOP trials (STOP error – STOP correct/STOP error + STOP correct).

We recorded from 809 VTA neurons from seven rats (1: 46 cells; 2: 141 cells; 3: 110 cells; 4: 137 cells; 5: 158 cells; 6: 120 cells; 7: 97 cells) during performance of a stop-change task (Fig. 1*A*,*B*), 85 of which were classified as being putative DA (see methods). The recording locations are illustrated in Figure 1*G*. We hypothesized that DA firing would reflect reward prediction error encoding, such that DA neurons would fire less to STOP cues, but more to STOP rewards. Consistent with this hypothesis, we found that many putative DA neurons fired more for GO cues over STOP cues during the response epoch, and more for STOP rewards over GO rewards during the reward epoch. This is illustrated in Figure 2*A–D*, which displays average firing of a single putative DA neuron aligned to port exit and reward delivery. The example neuron showed weaker firing on STOP trials after port exit during illumination of the STOP cue (Fig. 2*A*), and stronger firing on STOP trials at the time of reward delivery (Fig. 2*B–D*).

To determine whether DA neurons fired differently on STOP versus GO trials, we averaged firing rate across the 85 putative DA neurons and aligned activity to the initial GO cue (Fig. 2*E*), port exit (Fig. 2*F*), well entry (Fig. 2*G*), and reward delivery (Fig. 2*H*). During initial GO cue presentation, firing rate increased non-distinctly across all three trial types (Fig. 2*E*). After port exit, which is the time when the STOP cue was illuminated on STOP trials, firing appeared higher on GO (blue) trials compared to STOP (red) trials (Fig. 2*F*). We generated a trial-type index (STOP – GO/STOP + GO) for firing rates taken from the time of port exit to well entry (response epoch) on correct trials to determine whether the firing rate significantly differed between STOP and GO trials (Wilcoxon, *p* < 0.05). We found that the distribution of trial-type indices was significantly shifted below zero (Wilcoxon, µ = −0.02; *p* = 0.01; df = 84; Fig. 2*I*) and the counts of neurons that fired significantly more on GO trials outnumber those with the opposite effect (black bars; 3 STOP vs 11 GO; χ^2^ = 4.46; *p* = 0.03; Fig. 2*I*), suggesting that firing was stronger during the response epoch on GO versus STOP trials at both the population and single unit level.

Overall, firing appeared lower on STOP errors compared to correct STOP trials during the period following well entry (Fig. 2*G*). To quantify this effect, we computed an error index (STOP error – STOP correct/STOP error + STOP correct) on firing rates taken from the post-response epoch (800 ms after entering the fluid well) to determine whether the firing rate significantly differed between STOP correct and error trials across the entire population (Wilcoxon, *p* < 0.05). We found that the distribution was significantly shifted below zero (Wilcoxon, µ = −0.08; *p* = 0.009; df = 84; Fig. 2*J*) and the counts of neurons that fired significantly more on correct STOP trials outnumbers those that fired more on STOP errors (black bars; 8 STOP error vs 19 STOP correct; χ^2^ = 4.40; *p* = 0.04; Fig. 2*J*), suggesting that putative DA firing was stronger on correct STOP trials versus incorrect STOP trials after well entry.

Finally, we asked whether firing was higher during STOP trials when reward was delivered relative to GO trials by examining firing aligned to reward delivery (Fig. 2*H*). For this analysis, we excluded error trials from the alignment because reward is not delivered during incorrect trials. We found that firing was only slightly stronger on STOP compared to GO trials during the time of reward delivery. To quantify this effect, we computed the trial-type index (STOP – GO/STOP + GO) during the 500 ms period after reward was delivered (reward epoch). The distribution of indices was significantly shifted about zero, indicating that the number of neurons that fired more on STOP than GO trials were in the majority (Wilcoxon, µ = 0.02; *p* = 0.02; df = 84; Fig. 2*K*). Despite the significant positive shift in the population distribution, the counts of neurons that fired significantly more on STOP trials within a session was not greater than those that fired more on GO trials at the time of reward (black bars; 3 STOP vs 1 GO; χ^2^ = 0.90; *p* = 0.34; Fig. 2*K*). Overall these findings suggest that putative DA firing was modestly stronger on STOP versus GO trials at the time of reward delivery, suggesting that reward delivery after successful completion of a STOP trial elicited higher firing compared to rewards delivered after correct GO trials.

### Putative DA neurons fire less to STOP cues, but more for STOP rewards, when the rat responded more slowly

The degree of conflict associated with making the appropriate response varies from trial to trial during a session. One measure of how difficult it is to resolve conflict on any given trial is to determine how long rats take to successfully perform a STOP trial. That is, the more difficult the trial, the longer it takes a rat to inhibit and redirect behavior. To determine whether DA activity was modulated by the speed with which animals responded, average population histograms were split into fast and slow trials based on movement times within each session. To determine whether firing rate was significantly different between fast and slow trial types, we calculated speed indices on firing rates to compare fast and slow GO trials (GO fast – GO slow), fast and slow STOP trials (STOP fast – STOP slow), and fast and slow STOP errors (fast STOP error – slow STOP error). As before, we examined differences in firing across three behavioral epochs (response epoch, post-response epoch, and reward epoch).

**Figure 3.**
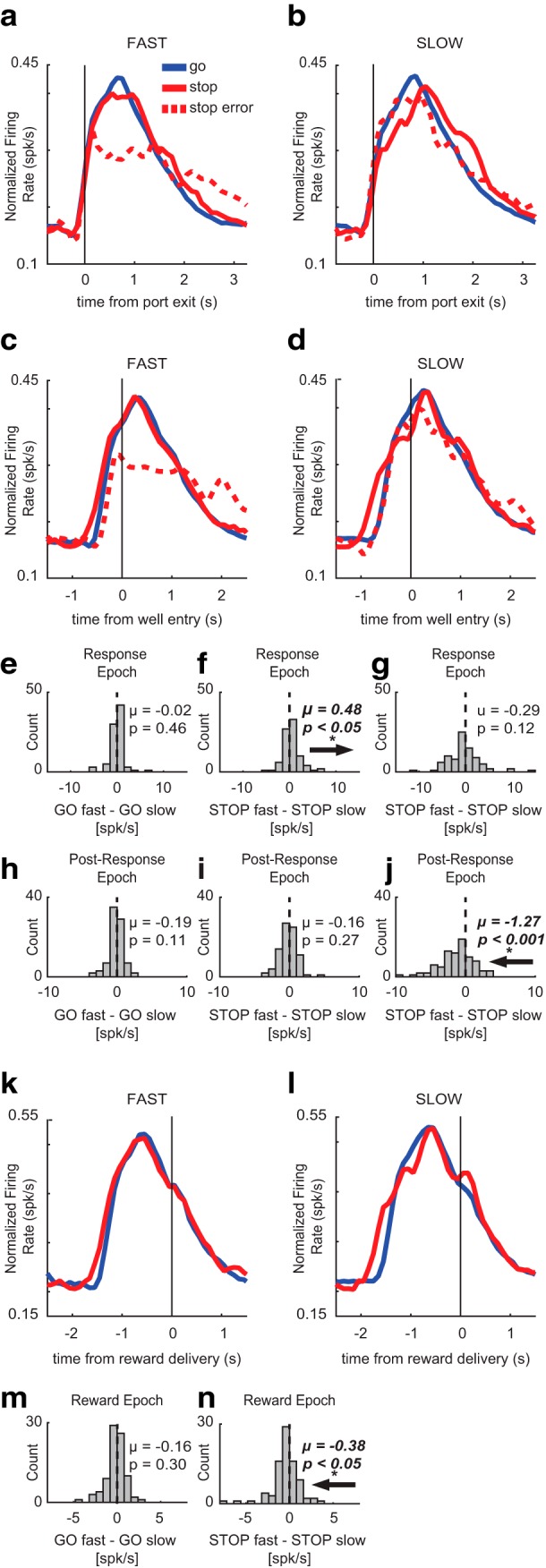
Movement time effects on putative DA firing. To determine whether putative DA activity was affected by speed of behavioral response, average population histograms were split into fast and slow trials based on movement time within each session and then averaged across sessions. ***A–D***, ***K***, ***L***, Average population histograms for fast and slow movement times for GO (blue), STOP (red), and STOP error (dashed) trials, aligned to port exit (***A***, ***B***), well entry (***C***, ***D***), and reward delivery (***K***, ***L***). ***E–J***, ***M***, ***N***, Population average distributions for effects between fast and slow trials. Arrows depict direction of distribution shift for significant effects. ***A***, ***B***, Activity was aligned to port exit for fast and slow movement times. ***E–G***, Firing rates were compared between fast and slow trial types during the response epoch by calculating trial type indices for GO trials (GO fast – GO slow), STOP trials (STOP fast – STOP slow), and STOP errors (STOP error fast – STOP error slow). During the response epoch, firing was significantly less on slow STOP trials compared to fast STOP trials (***F***), but there was no difference in firing between fast and slow GO trials (***E***) or STOP errors (***G***). ***C***, ***D***, Activity was aligned to well entry for fast and slow movement times. ***H***–***J***, Firing rates were compared between fast and slow trial types during the post-response epoch by calculating trial type indices described above. During the post-response epoch, firing on fast STOP errors was significantly lower than firing on slow STOP errors (***J***). There was no significant difference in firing between fast and slow GO (***H***) or STOP trials (***I***). ***K***, ***L***, Activity was aligned to reward delivery for fast and slow movement times. ***M***, ***N***, Firing rates were compared between fast and slow trials during the reward epoch by calculating trial type indices for GO trials (GO fast – GO slow) and STOP trials (STOP fast – STOP slow). During the reward epoch, firing was significantly higher on slow STOP trials compared to fast STOP trials (***N***). There was no significant difference between fast and slow GO trials at the time of reward (***M***).

Firing appeared higher on fast STOP trials compared to slow STOP trials during the response epoch (Fig. 3*A*,*B*). We found no significant differences in firing rate between fast and slow GO trials (Wilcoxon, µ = −0.02; *p* = 0.46; df = 84; Fig. 3*E*), or fast and slow STOP errors at the time of port exit (Wilcoxon, µ = −0.29; *p* = 0.12; df = 84; Fig. 3*G*). However, putative DA neurons fired significantly less on slow STOP trials compared to fast STOP trials after port exit (response epoch; Wilcoxon, µ = 0.48; *p* = 0.02; df = 84; Fig. 3*F*).

During the post-response epoch, firing appeared lower on fast STOP errors compared to slow STOP errors (Fig. 3*C*,*D*). After well entry, no significant differences in firing were apparent between fast and slow GO trials (Wilcoxon, µ = −0.19; *p* = 0.11; df = 84; Fig. 3*H*) or fast and slow STOP trials (Wilcoxon, µ = −0.16; *p* = 0.27; df = 84; Fig. 3*I*) were found. However, firing rates were significantly lower on fast STOP errors versus slow STOP errors during the post-response epoch (Wilcoxon, µ = −1.27; *p* < 0.001; df = 84; Fig. 3*J*), suggesting that putative DA neurons fired more on slower compared to faster STOP errors.

Lastly, we examined putative DA population aligned to reward delivery, where firing appeared to be higher on slow STOP rewards compared to fast STOP rewards (Fig. 3*K*,*L*). We found no significant differences in firing between fast and slow GO trials (Wilcoxon, µ = −0.16; *p* = 0.30; df = 84; Fig. 3*M*); however, DA neurons did fire significantly more during slower STOP trials compared to faster STOP trials (Wilcoxon, µ = −0.38; *p* = 0.04; df = 84; Fig. 3*N*).

### Putative DA neuron firing was modulated by heightened response conflict induced by previous trial type

**Figure 4.**
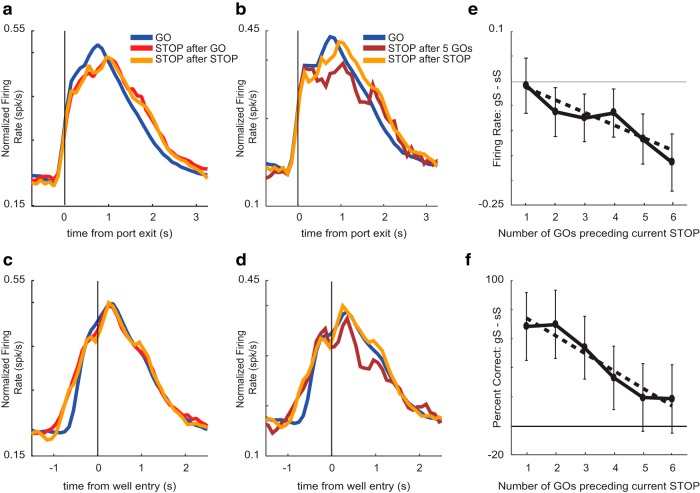
Effects of trial order on putative DA firing. Average putative DA activity (*n* = 85) plotted for GO trials, STOP trials preceded by a GO trial (gS), and STOP trials preceded by a STOP trial (sS). For reference, GO trials have the lowest conflict, while gS trials have the highest conflict. ***A***, ***C***, Average population histogram for GO trials (blue), gS (red), and sS (orange) trial types aligned to port exit (***A***) and well entry (***C***). ***B***, ***D***, Average population histogram for GO trials (blue), STOP trials after five GO trials (maroon), and sS trials (orange) aligned to port exit (***B***) and well entry (***D***). ***E***, To quantify effects, we computed indices on firing rates to compare sS to STOP trials with one to six previous GO trials (gS – sS/gS + sS) during the post-response epoch. ***F***, Percentage correct behavior on STOP trials preceded by one through six GO trials.

We investigated whether changes in difficulty induced by the previous trial modulated putative DA firing during performance on the current trial. Recall that rats perform better on STOP trials that followed a STOP trial (i.e., conflict adaptation; Fig. 1*F*). To determine whether the DA signal was impacted by the modulation of behavior due to the previous trial type, we examined average activity plotted on correct GO trials, STOP trials preceded by a single GO trial (gS), and STOP trials preceded by a STOP trial (sS). The average firing rate over time is illustrated to in Figure 4*A*,*C*. As described above, the average firing rate was higher on GO compared to STOP trials, but we found little difference between sS (orange) and gS (red) trials.

To quantify these effects, we computed indices on firing rates to compare gS to GO trials (gS – GO/gS + GO), sS to GO trials (sS – GO/sS + GO), and gS to sS trials (gS – sS/gS + sS) across the three behavioral epochs (response epoch, post-response epoch, and reward epoch). During the response epoch, firing on GO trials was significantly higher than both gS and sS trials (GO vs gS: Wilcoxon, µ = −0.04, *p* = 0.01, df = 84; GO vs sS: Wilcoxon, µ = −0.03, *p* = 0.01, df = 84). These findings demonstrate that putative DA firing is higher on low conflict GO trials compared to either gS or sS trials during the response epoch as described above; however, firing rates between sS and gS trials were not significantly different from each other in any of the analysis epochs (response epoch: Wilcoxon, µ = −0.004, *p* = 0.71, df = 84; post-response epoch: Wilcoxon, µ = −0.01; *p* = 0.43; reward epoch: Wilcoxon, µ = 0.01; *p* = 0.93) indicating that DA firing was not modulated on STOP trials by the nature of the previous trial (i.e., STOP or GO).

The lack of difference between sS and gS trials might reflect lack of encoding by DA neurons for this aspect of the task or that differences in difficulty between the two trial types was not strong enough to elicit differences in neural responding. To address this issue, we extended our analyses to study the effect that a train of multiple uninterrupted GO trials have on DA firing and percentage correct on STOP trials. Theoretically, the more GO trials that precede a STOP trial, the more difficult it would be to inhibit the response, thus lowering the probability of success. Indeed, we found a negative correlation between the number of previous GO trials and accuracy on the current STOP trial, such that STOP trial performance became worse with more preceding GO trials (*R*^2^ = 0.929; *p* = 0.002; Fig. 4*F*). Parallel to this result, we found significant reductions in firing as the number of previous GO trials increased. For example, Figure 4*B*,*D* illustrates firing on trials in which rats performed five GO trials before a successful STOP trial (5gS). Firing was significantly reduced on 5gS trials compared to 1gS trials (Wilcoxon, µ = −0.12, *p* < 0.05, df = 84).

To further quantify this effect, we computed indices to compare firing rates on sS trials and STOP trials preceded by multiple GO trials (gS – sS/gS + sS) ranging from one to six GO trials. We found a significant effect for the number of previous GO trials on the firing rate of the current STOP trial during the post-response epoch, where firing rate on STOP trials became lower as the number of preceding GO trials increased (*R*^2^ = 0.878; *p* = 0.006; Fig. 4*E*). Overall, these results suggest a positive relationship between performance and DA firing such that the worse rats were on gS trials, the lower firing should be. Indeed, we found a positive correlation between the two (*R*^2^ = 0.69; *p* = 0.04), demonstrating that lower probabilities of success were accompanied by reduced DA firing.

### Non-DA neurons fire more on STOP trials during the response period

**Figure 5.**
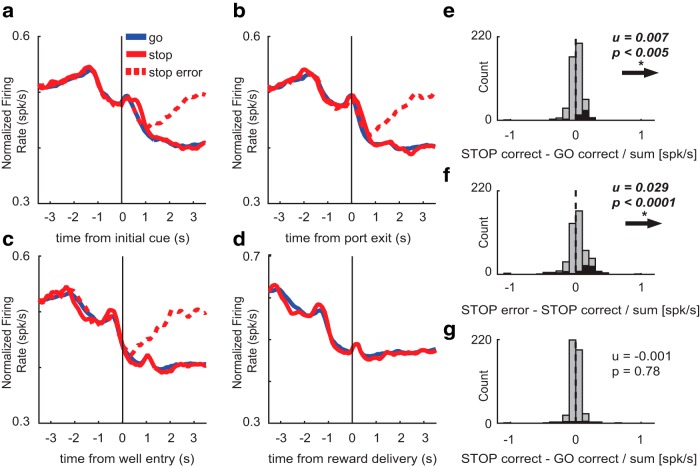
Characterization of non-DA firing. ***A–D***, Population average histograms of non-DA neurons plotted over trial time for GO (blue), STOP (red), and STOP error (dashed) trials while aligning to multiple events (*n* = 475). Activity is aligned to initial GO cue (***A***), port exit (***B***), well entry (***C***), and reward delivery (***D***). ***A***, At the time of the initial cue, firing rate increases for all trial types. ***B***, During the response epoch, firing was significantly higher on STOP trials compared to GO trials. ***C***, During the post-response epoch, firing was significantly higher on STOP errors compared to STOP corrects. ***D***, During the reward epoch, there was no significant difference in firing between STOP and GO trials. Error trials are excluded since reward is not delivered on errors. ***E–G***, Population average distributions for significant effects described above. Arrows depict direction of distribution shift for significant effects. Black bars represent the number of neurons that showed a significant difference between GO and STOP corrects (***E***, ***G***) and correct and incorrect STOP trials (***F***; Wilcoxon, *p* < 0.05). Distributions are determined to be significantly different from zero via Wilcoxon. ***E***, ***G***, Firing rate during the response and reward epoch were compared during correct STOPs and GOs by computing a trial type index (STOP correct – GO correct/STOP correct + GO correct). ***F***, Firing rate during the post-response epoch was compared in correct and incorrect STOP trials (STOP error – STOP correct/STOP error + STOP correct).

To determine whether firing patterns observed above were unique to putative DA neurons in VTA, we identically analyzed the 475 cells that were categorized as non-DA. Average firing over trial time aligned to multiple events is illustrated in Figure 5*A–D*. Firing of these neurons decreased on port entry and increased slightly at the time of GO cue presentation (Fig. 5*A*). Subsequently, after port exit, firing decreased on GO trials, but maintained a constant rate on STOP trials (Fig. 5*B*). Differences between trial types were not present when firing was aligned to well entry (Fig. 5*C*). On correct trials, firing remained low until the rat consumed the reward and exited the fluid well (data not shown). On error trials (dashed) firing decreased briefly and returned to baseline levels, again, at the time rats exited the fluid well (Fig. 5*C*, red dashed).

As above, we computed a trial type index (STOP – GO/STOP + GO) for firing rates taken from port exit to well entry (response-epoch) on correct trials to determine whether firing rate was significantly different between STOP and GO trials across the entire population (Wilcoxon, *p* < 0.05). The distribution was significantly shifted above zero (Wilcoxon, µ = 0.007; *p* < 0.005; df = 474; Fig. 5*E*) and the counts of neurons that fired significantly more on STOP trials outnumbered those with the opposite effect (black bars; 45 STOP vs five GO; χ^2^ = 31.84; *p* = 1.67^−8^; Fig. 5*E*), suggesting that firing was stronger on STOP versus GO trials during the response epoch at the population and single unit level. To quantify the difference between correct and error STOP trials, we generated the error index (STOP error – STOP correct/STOP error + STOP correct) on firing rates taken from the 800 ms period following well entry. During the post-response epoch, the distribution was significantly shifted above zero (Wilcoxon, µ = 0.029; *p* < 0.0001; Fig. 5*F*) and the counts of neurons that fired significantly less on STOP correct trials outnumbered those that fired more on STOP error trials (63 STOP error vs 19 STOP correct; χ^2^ = 23.50; *p* = 1.25^−6^; Fig. 5*F*, black bars). These results demonstrate that non-DA neurons fire more on STOP errors compared to correct STOP trials following well entry.

Lastly, we determined if firing of non-DA neurons would differ between GO and STOP trials at the time of reward (reward epoch; Fig. 5*D*) as they did for putative DA neurons. To quantify this effect, we computed the trial-type index (STOP – GO/STOP + GO) on firing rates taken during the 500 ms period following reward delivery (reward epoch). Non-DA neurons displayed no significant difference in firing between STOP and GO trials (Wilcoxon, µ = −0.002; *p* = 0.64; Fig. 5*G*) and the counts of neurons that fired more to rewards delivered on STOP trials did not differ significantly from the counts of neurons that fired more to rewards delivered on GO trials (15 STOP vs 9 GO; χ^2^ = 1.45; *p* = 0.23; Fig. 5*G*, black bars). Overall, these results demonstrate that non-DA cells fire opposite to that of putative DA cells during response and post-response epochs, and do not fire more on STOP trials during reward delivery as observed for putative DA neurons.

### Non-DA neuron firing is not modulated by movement speed

Next, we examined whether the speed of responding modulated the firing rate of non-DA neurons. As before, we generated average population histograms for both fast and slow trials based on movement time and examined differences in firing across three behavioral epochs (response epoch, post-response epoch, and reward epoch). To determine whether firing rate significantly differed between fast and slow trials, we calculated speed indices for firing rates on fast and slow GO trials (GO fast – GO slow), fast and slow STOP trials (STOP fast – STOP slow), and fast and slow STOP errors (fast STOP error – slow STOP error). Unlike the putative DA neurons, which fired differently on fast and slow STOP trials, non-DA neurons did not fire differently on fast and slow GOs, STOPs, or STOP errors during any epoch.

**Figure 6.**
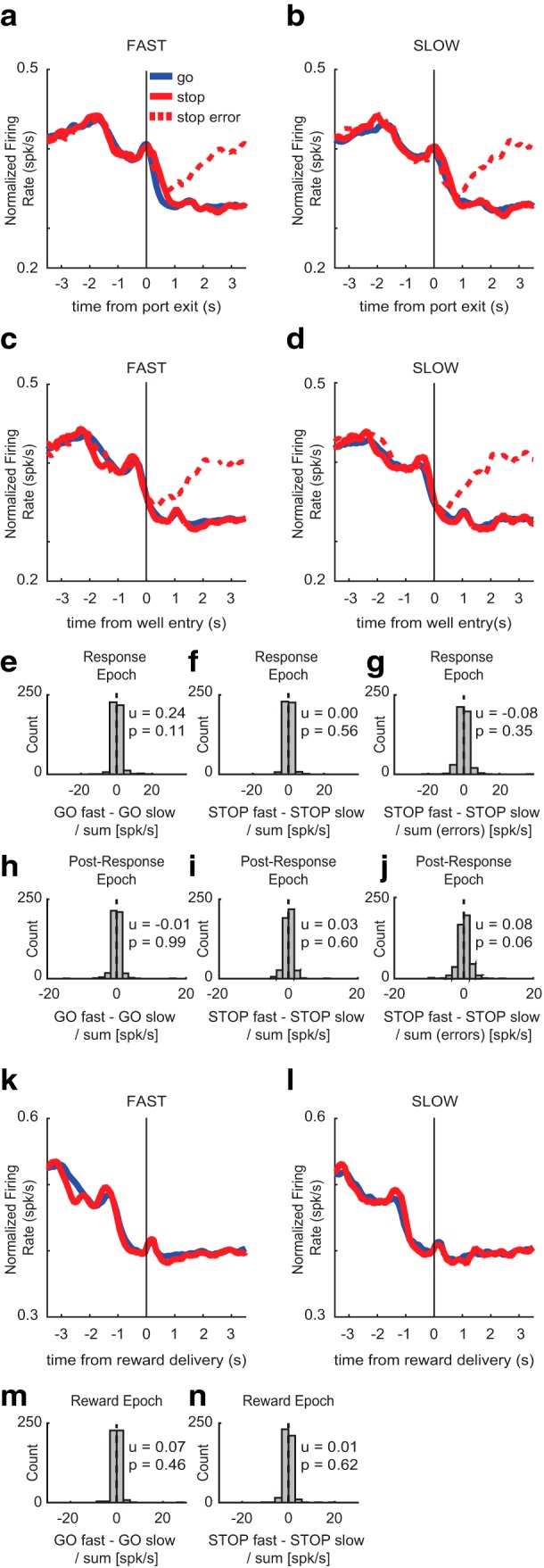
Movement time effects on non-DA firing. To determine whether non-DA activity was affected by speed of behavioral response, average population histograms were split into fast and slow trials based on movement time. ***A–D***, ***K***, ***L***, Average population histograms for fast and slow movement times for GO (blue), STOP (red), and STOP error (dashed) trials, aligned to port exit (***A***, ***B***), well entry (***C***, ***D***), and reward delivery (***K***, ***L***). ***A***, ***B***, Activity was aligned to port exit for fast and slow movement times. ***E–G***, Firing rates were compared between fast and slow trial types during the response epoch by calculating trial type indices for GO trials (GO fast – GO slow/GO fast + GO slow), STOP trials (STOP fast – STOP slow/STOP fast + STOP slow), and STOP errors [STOP fast – STOP slow/STOP fast + STOP slow (errors)]. ***C***, ***D***, Activity aligned to well entry for fast and slow movement times. ***H–J***, Firing rates were compared between fast and slow trial types during the post-response epoch using indices described above. ***K***, ***L***, Activity was aligned to reward delivery for fast and slow movement times. ***M***, ***N***, Firing rates were compared between fast and slow trials during the reward epoch by calculating the same trial-type indices described above.

During the response epoch, we observed no apparent differences between the firing of non-DA cells on fast and slow trial types (Fig. 6*A*,*B*). We found no significant differences in firing between fast and slow GO trials (Wilcoxon, µ = 0.24; *p* = 0.11; df = 474; Fig. 6*E*), fast and slow STOP trials (Wilcoxon, µ = 0.00; *p* = 0.56; df = 474; Fig. 6*F*), or fast and slow STOP error trials (Wilcoxon, µ = −0.08; *p* = 0.35; df = 474; Fig. 6*G*). During the post-response epoch, firing appeared to be the same for fast and slow trial types (Fig. 6*C*,*D*). There were no significant differences between fast and slow GO trials (Wilcoxon, µ = −0.01; *p* = 0.99; df = 474; Fig. 6*H*), fast and slow STOP trials (Wilcoxon, µ = 0.03; *p* = 0.60; df = 474; Fig. 6*I*), or fast and slow STOP error trials (Wilcoxon, µ = 0.08; *p* = 0.06; df = 474; Fig. 6*J*) during the post-response epoch. Lastly, during the reward epoch, firing appeared to be the same between fast and slow GO and STOP trials (Fig. 6*K*,*L*). There was no significant difference between fast and slow GO trials (Wilcoxon, µ = 0.07; *p* = 0.46; Fig. 6*M*), or fast and slow STOP trials (Wilcoxon, µ = 0.01; *p* = 0.62; df = 474; Fig. 6*N*) at the time of reward.

### Non-DA neuron firing was modulated by heightened response conflict induced by previous trial type

**Figure 7.**
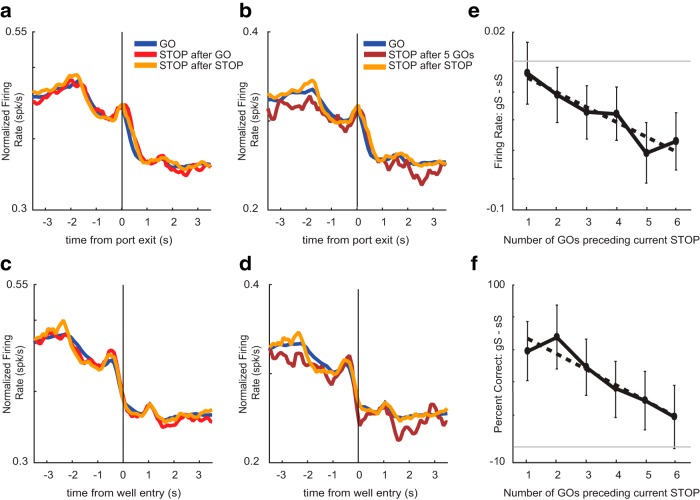
Effects of trial order on non-DA firing. Average non-DA activity (*n* = 475) plotted for GO trials, STOP trials preceded by a GO trial (gS), and STOP trials preceded by a STOP trial (sS). ***A***, ***C***, Average population histogram for GO trials (blue), gS (red), and sS (orange) trial types aligned to port exit (***A***) and well entry (***C***). ***B***, ***D***, Average population histogram for GO trials (blue), STOP trials after five GO trials (maroon), and sS trials (orange) aligned to port exit (***B***) and well entry (***D***). ***E***, To quantify effects, we computed indices on firing rates to compare sS to STOP trials with one to six previous GO trials gS to GO trials (gS – GO/gS + GO) during the post-response epoch. ***F***, Percentage correct behavior on STOP trials preceded by one through six GO trials.

Lastly, we investigated whether the previous trial type would affect non-DA firing on the current trial. To determine whether non-DA cells might contribute to behavior modulation based on previous trial type, we compared average firing activity on correct GO trials, STOP trials preceded by up to six preceding GO trials (gS), and STOP trials following a STOP trial (sS). A regression analysis comparing percentage correct between the six trial types revealed a significant effect between the number of previous GO trials and accuracy on the current STOP trial during sessions during which non-DA neurons were recorded (*R* ^2^ = 0.898; *p* = 0.004; Fig. 7*F*). We also found that firing on current STOP trials was modulated by the number of preceding GO trials. As an example, we plotted firing on STOP trials preceded by five GO trials (Fig. 7*B*,*D*). As before, this effect was quantified by computing indices to compare firing rates between sS trials and STOP trials preceded by multiple GO trials (gS – sS/gS + sS) during the post-response epoch (Fig. 7*E*). During the post-response epoch, we found a significant effect for the number of previous GO trials on firing rate of the current STOP trial, such that firing rate on STOP trials became lower as the number of preceding GO trials increased (*R* ^2^ = 0.887; *p* = 0.005).

## Discussion

In this study, we recorded activity of putative DA neurons in the VTA from rats performing a stop-change task. Phasic bursts in DA have been shown to reflect both reward prediction errors and saliency in a variety of tasks, yet it is unclear how DA firing is modulated as a function of performance on tasks that require response inhibition and cognitive control. On the one hand, if DA activity reflects changes in value associated with a particular cue, we would expect to see decreased DA firing at the time of cues that predict a lower probability of receiving reward (i.e., STOP cues), followed by elevated DA firing at the time of unexpected reward. In this light, DA activity can be thought of as an indirect indicator of an animal’s intuition about its probability of success on a given trial. In support of this hypothesis, previous literature has shown that DA neurons encode reward probabilities and outcomes, such that cues predicting a lower probability of reward yield weaker phasic DA responses, while unexpected and low probability rewards yield phasic increases in DA activity (Fiorillo et al., 2003). On the other hand, if DA activity reflects saliency or the need to inhibit behavior, we would expect to see increased firing of DA neurons to STOP cues.

We found very few DA neurons that fired significantly stronger on STOP trials during the response epoch, and that firing to STOP cues was dependent on the identity of the previous trial type, in agreement with the behavioral evidence for heightened response conflict. We found that across the population and at the single neuron level, putative DA neurons exhibited lower and higher firing to STOP cues and rewards, respectively. This prediction error effect was enhanced as the number of preceding GO trials increased, such that DA’s activity was modulated by the conflict associated with an unexpected STOP trial following a train of GO trials. These findings support our idea that DA signaling on the stop-change task is indicative of an animal’s sense about its future probability of success, and are also in line with previously published work linking the activity of midbrain DA neurons to an animal’s belief in choice accuracy during a perceptual decision-making task (Lak et al., 2017). In that study, using a computational modeling approach, DA neurons were shown to be sensitive to reward prediction error and the same signal also represented statistical certainty in reward (Lak et al., 2017).

In many ways, these findings are supported by other studies that suggest two distinct populations of DA neurons exist (Matsumoto and Hikosaka, 2009). Recordings from substantia nigra pars compacta (SNc) reveal a gradient in the density of neurons encoding motivational value versus saliency with greater numbers of value encoding neurons found along the ventromedial extent (Matsumoto and Hikosaka, 2009). This is somewhat in contrast with the VTA, where high numbers of value encoding neurons have been described, along with sparse numbers of saliency encoding neurons (Matsumoto and Hikosaka 2009, Bromberg-Martin et al., 2010). These findings, originally described in non-human primates, largely fit with the results we present here, and suggests that DA neurons in SNc might report the salience of stop cue during performance of our stop-change task.

Previously, pharmacologic control of response inhibition via the DA system has yielded conflicting results on DA’s role in GO and STOP trial performance (Eagle and Baunez, 2010). In children and adults with ADHD, administration of methylphenidate and D-amphetamine, psychostimulants that target the DA system, have reported improvement in the stop signal reaction time (SSRT), a common measure of response inhibition in which the longer the SSRT the more time the animal needs to inhibit the response (Tannock et al., 1989; Aron et al., 2003; Boonstra et al., 2005). However, other studies using the same psychostimulants in hyperactive children report improvements on GO reaction time but not SSRT, as well as other forms of unwanted impulsivity (Bedard et al., 2003; Lijffijt et al., 2006). Still, there is evidence that DA’s effects may be baseline dependent, decreasing SSRT in slow responders, and increasing or worsening SSRT, in subjects with fast SSRTs (Boonstra et al., 2005; Eagle et al., 2007). Collectively, these results suggest that systemic increases in DA may non-selectively enhance either GO or STOP performance, which may be dependent on the subject’s baseline performance at the start of the experiment. DA’s role in action control may be better explained at the receptor level. A study that administered GBR-12909, a DA reuptake inhibitor, reported no effect on SSRT in rats (Bari et al., 2009). Administration of cis-flupenthixol, a mixed D1/D2 receptor antagonist, also had no impact on SSRT in rats, and failed to block the SSRT-decreasing effect of methylphenidate (Eagle et al., 2007). However, administration of either a D1- or D2-receptor antagonist (SCH23390 or sulpiride) directly into the dorsal medial striatum (DMS) led to opposing effects on SSRT, with SSRT decreasing after D1 antagonism, and increasing after D2 antagonism (Eagle and Baunez, 2010). While the DMS is well-characterized regarding its role in decision-making and action selection and initiation, further research is needed to assess how the DMS decodes midbrain DA input in the context of the stop-signal task.

The nucleus accumbens (NAc) also receives DA projections and plays a key role in reward-seeking and impulsivity. In one study, the depletion of DA in the NAc attenuated amphetamine-induced increases in premature responding during a 5-choice serial reaction time task (5-CSRTT; Cole and Robbins, 1989). Remarkably, amphetamine-induced increases in premature responding could be blocked by systemic administration of D1/D2 mixed receptor antagonist, cis-flupenthixol, and D1 receptor antagonist, SCH23390, although these antagonists had no impact on SSRT (Eagle and Baunez, 2010). Increased impulsivity is also associated with reduced D2/D3 receptor activity in the NAc (Eagle and Baunez, 2010; Dalley and Robbins, 2017). These findings suggest that NAc DA transmission may modulate impulsivity via receptor-mediated processes. There is also evidence suggestingt that NAc DA release is modulated by action initiation. In a study that measured DA concentration in freely-behaving rats during a go/no-go task, NAc DA increased on no-go trials only after correct movement was initiated for this trial type (Syed et al., 2016). These findings are suggestivet that downstream consequences of DA in higher level processing areas are critical for task performance.

The role of DA in the regulation of impulsivity is largely dependent on the type of impulsivity being tested (Dalley and Robbins, 2017). Waiting impulsivity, an animal’s ability to refrain from responding until receiving a specific cue or amount of time has elapsed, is thought to be influenced by the activity of DA cells arising from the VTA that project to the ventral striatum. However, response inhibition or “stopping impulsivity,” an animal’s ability to stop and redirect a prepotent action, is largely dependent on the action of DA in the dorsal striatum (Dalley and Robbins, 2017). It is unclear to what degree DA from VTA neurons influence or support response inhibition. Since DA neurons in VTA strongly project to NAc, we suspect the role that VTA DA neurons have on stop-signal performance is to track the probability of reward, as opposed to signaling saliency or the need to inhibit behavior. As suggested above, if we were to record from SNc that contains DA neurons projecting strongly to dorsal striatum, we might observe a higher percentage of neurons that fire more strongly to STOP cues, thus playing a more direct role in response inhibition.

Critically, we do not see similar changes in non-DA neurons. In the main analyses we found that non-DA cells in the VTA actually showed the opposite effect; higher firing on STOP trials compared to GO trials. However, this effect might simply reflect differences in when the movement was terminated, because firing did not increase above GO-induced firing but simply extended until the end of the movement. Non-DA cells did, however, exhibit sequence effects that mirrored those seen in DA cells such that increased numbers of GO trials before a STOP trial resulted in reduced activity and a decreased likelihood of responding correctly. These signals may reflect reward expectancy signals either in the form of future motor planning events or being reflective of the overall integration events occurring in VTA (Watabe-Uchida et al., 2017).

The stop-change task requires animals to inhibit a GO response in the presence of a STOP cue and the realization and utilization of this strategy is integral to successful performance. In the goal-directed behavior literature, there is new debate surrounding the involvement of DA in the interplay between model-based and model-free behaviors (Langdon et al., 2018). Phasic bursts in DA have traditionally been interpreted as model-free generated prediction errors as an animal encounters valuable information or reward (Langdon et al., 2018). In our task, increases in DA to reward on STOP trials could reflect general learning or “surprise” associated with receiving reward. However, given that these increases in DA firing to reward also occurred on trials in which rats adaptively slowed their behavior, these changes in DA firing may be reflective of the rats adopting a model-based approach. There is reason to think that DA signals are heterogeneous, and not simply scalar representations of value independent of the form of the expected reward (Sadacca et al., 2016; Soares et al., 2016; Starkweather et al., 2017; Langdon et al., 2018). If true, components of the overall DA response may reflect model-free and model-based predictions. Future research should explore the specific temporal components of this response to further elucidate the exact computational support VTA DA cells are offering neural networks supporting response inhibition.

Response inhibition is a complex and dynamic behavior that is reliant on several brain regions. In our study, we show that DA signaling in VTA neurons appears to reflect the uncertainty associated with a low probability of reward on STOP trials. This is distinct from commonly posited beliefs that VTA DA signaling is necessary to engage in response inhibition or provides a neural correlate of saliency associated with the low occurrence of STOP trials. These data are some of the first to characterize a function for VTA DA neurons during a task that requires response inhibition.

## Synthesis

Reviewing Editor: Philippe Tobler, University of Zurich Decisions are customarily a result of the Reviewing Editor and the peer reviewers coming together and discussing their recommendations until a consensus is reached. When revisions are invited, a fact-based synthesis statement explaining their decision and outlining what is needed to prepare a revision will be listed below. The following reviewer(s) agreed to reveal their identity: Benjamin Saunders, Armin Lak

The authors report the results of electrophysiological recording experiments in putative midbrain dopamine (DA) neurons of the ventral tegmental area, while rats perform a stop-signal task, where an initial sensory cue instructs ‘Go’ while a second sensory cue (appearing in 20% of trials) instructs “Stop and go to the opposite side ”. Putative DA neurons from this recording dataset showed higher firing rates in response to GO trial cues, relative to STOP trial cues, and less firing on error trials. The findings show that neuronal responses are overall consistent with prediction error coding, rather than encoding the saliency of rare ‘Stop’ trials and suggests that they encode something about the uncertainty of the task, along the lines of reward prediction error models.

Both reviewers found potential merit in the manuscript, which offers insights into DA responses in a behavioural task that has been mainly used in pharmacological studies of dopamine and less in electrophysiological experiments. However, they also noted that the differences in neuronal responses seem mild, the manuscript and raised several concerns which need to be thoroughly addressed in a revision.

Major concerns:

1. The differences in the neuronal activity in various trials types seem rather mild. As such, it would be useful if the manuscript could include few analysis that further establish basic responses of these neurons. For instance, it would be useful if we could see responses of example neurons (similar to Fig 2a-b) to reward/no reward in a short time window around the outcome time.

2. The manuscript proposes two different hypotheses (Line 78-81): value-based prediction error (PE) coding vs encoding the saliency of rare Stop trials independent of value. The manuscript comes back to these two hypotheses in the discussion to suggest that neuronal responses are consistent with the PE coding (particularly with a variant of PE coding that includes perceptual uncertainty as described in Lak et al 2017). While this has been described in the manuscript, it would be best if authors could further spell out their discussion about these concepts (in the first and second paragraphs in the discussion) to avoid any confusion. Current discussion paragraphs might make the impression that the results are not consistent with Fiorillo et al., 2003. However, the results are consistent with PE coding shown in Fiorillo et al., 2003. It is however the case that due to the perceptual uncertainty inherent in the task (as well as outcomes being contingent on choices), variants of PE coding that include perceptual uncertainty and choice (Lak et al 2017) could be used to interpret the data.

3. The authors argue that DA neurons do not encode the saliency of STOP cues, but does the greater firing to GO trial cues reflect the greater saliency (they occur more frequently) and/or motivational value (they more often predict reward) of GO trial cues?

4. Conditioning DA responses on previous trial type (Figure 4) seems to not show any significant effect. One wonders if significant effects could come out if the authors consider more than 1 trial back in their analysis. Ideally they could use a regression model that includes few past trials (similar to those used previously by Bayer and Glimcher 2005 and others).

5. Given that only 85 of the 809 recorded neurons were determined to be putative DA cells (and therefore the only ones presented in the paper), it seems like a there is a lot of richness in this dataset that could be explored upon revision. One wonders about the activity of putative non-DA neurons in this task. Do they have different firing patterns with respect to trial type and reward exposure, or some other variable, compared to putative DA neurons. It would be interesting, and extend the importance of this paper, if divergent encoding was found for non-DA versus putative DA cells. If the putative non-DA cells show similar results, that would be a surprising and interesting finding too. Either way, there is a lot of untapped information here that could broaden the paper's scope in a useful way (presumably with relatively low effort on behalf of the authors, given that it sounds like all the data for these neurons has been collected). There is little midbrain recording data in a task like this in general, and very little on midbrain non DA neurons. Moreover, such an analysis would allow the authors to connect their results with a lot of the recent midbrain heterogeneity work from Uchida and other groups.

The reviewers are aware that technical reasons such as electrode drift might not have allowed authors to hold many of the neurons for long enough (this issue is common among many e-phy recordings, specially in chronic recordings from freely moving animals, and is not specific to this study). As such, it is conceivable that the included neurons did not only meet waveform criteria but were also recorded for long enough, and similarly, many of the neurons which were not included in the study were also those that were not recorded for long enough. In such a situation having a conservative criterion would allow to classify some of the neurons as DA neurons but the remaining neurons would be a mixture of DA and non-DA neurons. Authors might be able to go beyond this and redo their cell classification and divide them into 3 groups of DA, non-DA and non-classified. In that case, they will need to substantially expand on their classification methods, single unit quality and recording stability.

Intermediate and minor points

1. General comment on results: there are several errors or lack of text references to the specific panels being talked about in the figures, which makes reading of the results difficult.

2. Line 215: Figure 2b: the claim that DA responses to reward are larger after Stop compared to Go is not visually clear in the example neuron. It might be useful to change the x-axis to not include preceding cue responses so that the reader could focus on the reward responses. Making separate panels for cue and reward responses would also help.

3. Is the time of the second cue (in Stop trials) the same as the time of port exit? If yes, this should be stated. If not, the authors may want to align DA responses to the second cue.

4. The reporting of the behavioral data in Figure 1 is a little unclear. Which sessions are these data points from? At the bottom of page 8, the authors refer to “a two-way ANOVA across cells”, what cells does this refer to and which data points in the figure, or is it a typo?

5. Please indicate statistically significant effects on the figures with an asterisk of some other symbol.

6. Figure 2b caption reference to the left and right raster plots needs to be reversed.

7. Figure 2c is not described anywhere in the text.

8. For all the bar histogram plots it would be helpful to the reader if the direction of the distribution shift were labelled, maybe with an arrow on the panel. Since there are a lot of them, this will help quickly figure out which are significant, and how.

9. It might be possible to provide a title that gives the reader in a declarative statement a more direct idea of the findings here. This is really just a style suggestion, not a criticism

10. Typo: line 189: rat performed exhibited.

## Author Response

Synthesis Statement for Author (Required):

The authors report the results of electrophysiological recording experiments in putative midbrain dopamine (DA) neurons of the ventral tegmental area, while rats perform a stop-signal task, where an initial sensory cue instructs ‘Go’ while a second sensory cue (appearing in 20% of trials) instructs “Stop and go to the opposite side ”. Putative DA neurons from this recording dataset showed higher firing rates in response to GO trial cues, relative to STOP trial cues, and less firing on error trials. The findings show that neuronal responses are overall consistent with prediction error coding, rather than encoding the saliency of rare ‘Stop’ trials and suggests that they encode something about the uncertainty of the task, along the lines of reward prediction error models.

Both reviewers found potential merit in the manuscript, which offers insights into DA responses in a behavioural task that has been mainly used in pharmacological studies of dopamine and less in electrophysiological experiments. However, they also noted that the differences in neuronal responses seem mild, the manuscript and raised several concerns which need to be thoroughly addressed in a revision.

RESPONSE: We thank the reviewers for their thoughtful comments. We have taken them seriously and have addressed all of them below.

Major concerns:

1. The differences in the neuronal activity in various trials types seem rather mild. As such, it would be useful if the manuscript could include few analysis that further establish basic responses of these neurons. For instance, it would be useful if we could see responses of example neurons (similar to Fig 2a-b) to reward/no reward in a short time window around the outcome time.

RESPONSE: We have now zoomed in on the reward period during correct GO and STOP trials in Figure 1 and now show correct (reward) and incorrect STOP trials (no reward) as requested in the single cell example. Population histograms also show correct and error trials. In addition we did a basic analysis describing counts of neurons that were active during the response period. Of the 85 reward responsive putative DA neurons, 77 were also responsive during the response epoch. Further we have analyzed non-DA firing and now show that the responses of putative DA neurons are unique.

2. The manuscript proposes two different hypotheses (Line 78-81): value-based prediction error (PE) coding vs encoding the saliency of rare Stop trials independent of value. The manuscript comes back to these two hypotheses in the discussion to suggest that neuronal responses are consistent with the PE coding (particularly with a variant of PE coding that includes perceptual uncertainty as described in Lak et al 2017). While this has been described in the manuscript, it would be best if authors could further spell out their discussion about these concepts (in the first and second paragraphs in the discussion) to avoid any confusion. Current discussion paragraphs might make the impression that the results are not consistent with Fiorillo et al., 2003. However, the results are consistent with PE coding shown in Fiorillo et al., 2003. It is however the case that due to the perceptual uncertainty inherent in the task (as well as outcomes being contingent on choices), variants of PE coding that include perceptual uncertainty and choice (Lak et al 2017) could be used to interpret the data.

RESPONSE: We apologize for the lack of clarity. We did not mean to give the impression that the results are not consistent with Fiorillo et al. We have revamped the section describing PE and saliency encoding in the discussion paragraphs. We have further described our results in the framework of PE and saliency encoding, paying close attention to whether we expect to see an increase or decrease in putative-DA activity at the time of cue and reward presentation.

3. The authors argue that DA neurons do not encode the saliency of STOP cues, but does the greater firing to GO trial cues reflect the greater saliency (they occur more frequently) and/or motivational value (they more often predict reward) of GO trial cues?

RESPONSE: We think that the repetitive and frequent nature of GO trials in comparison to STOP trials makes them redundant and less salient than STOP trials. Since GO trials occur on 80% of trials they are highly expected and rats become very automatic at the task. Previous work has shown that oddball, unexpected or incongruent events appear more salient. For example, pupil dilation is modulated when monkey experience high conflict trials (Ebitz et al).

4. Conditioning DA responses on previous trial type (Figure 4) seems to not show any significant effect. One wonders if significant effects could come out if the authors consider more than 1 trial back in their analysis. Ideally they could use a regression model that includes few past trials (similar to those used previously by Bayer and Glimcher 2005 and others).

RESPONSE: This is a terrific suggestion. Thank you. To address this issue, we extended our analysis to include multiple past trials in order to compare putative DA firing on STOP trials preceded by one through six GO trials. We found that percent correct and putative DA firing gradually became lower on the current STOP trial as it was preceded by more GO trials. In a final analysis we show a significant correlation between percent correct and firing rate against number of GOs that preceded a successful STOP trial and a direct correlation between firing and percent correct.

-

5. Given that only 85 of the 809 recorded neurons were determined to be putative DA cells (and therefore the only ones presented in the paper), it seems like a there is a lot of richness in this dataset that could be explored upon revision. One wonders about the activity of putative non-DA neurons in this task. Do they have different firing patterns with respect to trial type and reward exposure, or some other variable, compared to putative DA neurons. It would be interesting, and extend the importance of this paper, if divergent encoding was found for non-DA versus putative DA cells. If the putative non-DA cells show similar results, that would be a surprising and interesting finding too. Either way, there is a lot of untapped information here that could broaden the paper's scope in a useful way (presumably with relatively low effort on behalf of the authors, given that it sounds like all the data for these neurons has been collected). There is little midbrain recording data in a task like this in general, and very little on midbrain non DA neurons. Moreover, such an analysis would allow the authors to connect their results with a lot of the recent midbrain heterogeneity work from Uchida and other groups.

RESPONSE: Thanks for the suggestion. We agree. We have replicated the DA analysis in non-DA neurons. The majority of effects that we described for DA neurons were not present in the non-DA population. Non-DA neurons were not modulated by movement speed suggesting that degree of conflict present on a given set of trials did not impact firing in these neurons. For the main analysis we see effects opposite DA neurons. Specifically higher firing on STOP relative to GO trials. However, firing on STOP trials does not exceed what is present during presentation of the GO cue and differences between STOP and GO trials disappear when activity is aligned to well entry (i.e., termination of the movement). The full analysis is now described in the results section and we have added 3 new figures illustrating the results. With regards to the Uchida suggestion we agree, and included some of our thoughts in the Discussion.

Intermediate and minor points

1. General comment on results: there are several errors or lack of text references to the specific panels being talked about in the figures, which makes reading of the results difficult.

RESPONSE: We apologize. We now do a better job referencing panels and hopefully corrected all errors.

2. Line 215: Figure 2b: the claim that DA responses to reward are larger after Stop compared to Go is not visually clear in the example neuron. It might be useful to change the x-axis to not include preceding cue responses so that the reader could focus on the reward responses. Making separate panels for cue and reward responses would also help.

RESPONSE: As requested we have added panels to the figure that focus on reward-related activity without preceding cue responses.

3. Is the time of the second cue (in Stop trials) the same as the time of port exit? If yes, this should be stated. If not, the authors may want to align DA responses to the second cue.

RESPONSE: We now state that STOP cue onset and port exit is simultaneous in the figure. In general, we tend not to align to the STOP cue because the goal of most analyses is to compare GO versus STOP trials.

4. The reporting of the behavioral data in Figure 1 is a little unclear. Which sessions are these data points from? At the bottom of page 8, the authors refer to ‘a two-way ANOVA across cells’, what cells does this refer to and which data points in the figure, or is it a typo?

RESPONSE: We now clearly state data was analyzed using a two-way ANOVA, where each datum is a session average.

5. Please indicate statistically significant effects on the figures with an asterisk of some other symbol.

RESPONSE: We have added arrows and asterisks. Thank you. The figures are more clear with these additions.

6. Figure 2b caption reference to the left and right raster plots needs to be reversed.

RESPONSE: We have reversed them.

7. Figure 2c is not described anywhere in the text.

RESPONSE: We now describe figure 2c.

8. For all the bar histogram plots it would be helpful to the reader if the direction of the distribution shift were labelled, maybe with an arrow on the panel. Since there are a lot of them, this will help quickly figure out which are significant, and how.

RESPONSE: We have added arrows and asterisks. Thank you. The figures are more clear with these additions.

9. It might be possible to provide a title that gives the reader in a declarative statement a more direct idea of the findings here. This is really just a style suggestion, not a criticism

RESPONSE: We agree. We have changed that title to ‘: Firing of putative dopamine neurons in ventral tegmental area is modulated by probability of success during performance of a stop-change task’

10. Typo: line 189: rat performed exhibited.

RESPONSE: Corrected. Thanks.
